# What is needed to implement a computer-assisted health risk assessment tool? An exploratory concept mapping study

**DOI:** 10.1186/1472-6947-12-149

**Published:** 2012-12-19

**Authors:** Farah Ahmad, Cameron Norman, Patricia O’Campo

**Affiliations:** 1 School of Health Policy and Management, York University, 4700 Keele Street, HNES Building, 4th Floor, Toronto, ON, M3J 1P3, Canada; 2 Dalla Lana School of Public Health, University of Toronto, Toronto, Canada; 3 Centre for Research on Inner City Health, The Keenan Research Centre in the Li Ka Shing Knowledge Institute, St. Michael's Hospital, Toronto, Canada; 4 CENSE Research + Design, Toronto, ON, Canada

**Keywords:** Computer-assisted, Risk assessment, Psychosocial health, Decision-support, Qualitative, Canada, Underserved urban population

## Abstract

**Background:**

Emerging eHealth tools could facilitate the delivery of comprehensive care in time-constrained clinical settings. One such tool is interactive computer-assisted health-risk assessments (HRA), which may improve provider-patient communication at the point of care, particularly for psychosocial health concerns, which remain under-detected in clinical encounters. The research team explored the perspectives of healthcare providers representing a variety of disciplines (physicians, nurses, social workers, allied staff) regarding the factors required for implementation of an interactive HRA on psychosocial health.

**Methods:**

The research team employed a semi-qualitative participatory method known as Concept Mapping, which involved three distinct phases. First, in face-to-face and online brainstorming sessions, participants responded to an open-ended central question: “What factors should be in place within your clinical setting to support an effective computer-assisted screening tool for psychosocial risks?” The brainstormed items were consolidated by the research team. Then, in face-to-face and online sorting sessions, participants grouped the items thematically as ‘it made sense to them’. Participants also rated each item on a 5-point scale for its ‘importance’ and ‘action feasibility’ over the ensuing six month period. The sorted and rated data was analyzed using multidimensional scaling and hierarchical cluster analyses which produced visual maps. In the third and final phase, the face-to-face Interpretation sessions, the concept maps were discussed and illuminated by participants collectively.

**Results:**

Overall, 54 providers participated (emergency care 48%; primary care 52%). Participants brainstormed 196 items thought to be necessary for the implementation of an interactive HRA emphasizing psychosocial health. These were consolidated by the research team into 85 items. After sorting and rating, cluster analysis revealed a concept map with a seven-cluster solution: 1) the HRA’s equitable availability; 2) the HRA’s ease of use and appropriateness; 3) the content of the HRA survey; 4) patient confidentiality and choice; 5) patient comfort through humanistic touch; 6) professional development, care and workload; and 7) clinical management protocol. Drawing insight from the theoretical lens of Sociotechnical theory, the seven clusters of factors required for HRA implementation could be read as belonging to three overarching aspects : Technical (cluster 1, 2 and 3), Social-Patient (cluster 4 and 5), and Social-Provider (cluster 6 and 7). Participants rated every one of the clusters as important, with mean scores from 4.0 to 4.5. Their scores for feasibility were somewhat lower, ranging from 3.4 to. 4.3. Comparing the scores for importance and feasibility, a significant difference was found for one cluster from each region (cluster 2, 5, 6). The cluster on professional development, care and workload was perceived as especially challenging in emergency department settings, and possible reasons were discussed in the interpretation sessions.

**Conclusion:**

A number of intertwined multilevel factors emerged as important for the implementation of a computer-assisted, interactive HRA with a focus on psychosocial health. Future developments in this area could benefit from systems thinking and insights from theoretical perspectives, such as sociotechnical system theory for joint optimization and responsible autonomy, with emphasis on both the technical and social aspects of HRA implementation.

## Background

In the past century, health and healthcare have increasingly become understood as multifaceted. In population health sciences, including health promotion, the socio-ecological model of health has extended the lens of inquiry regarding illness factors beyond the body to proximate and distal factors in social and physical environments. In clinical practice, two separate but related concepts have been put forth to aid practitioners in addressing the multifaceted nature of health. The first is *co-morbidity*, introduced in the 1970s by Alvin R. Feinstein to refer to “any distinct additional entity that has existed or may occur during the clinical course of a patient who has the index disease under study” [1]. This gained rapid attention among health practitioners, policy makers and researchers due to increasing life expectancy and technological advances in diagnosis and treatment of co-morbid conditions [2]. Yet, the concept of co-morbidity gives inadequate attention to patient’s life context without which better health outcomes might not be attainable. Recently, Valders and colleagues identified the *patient complexity* as an important factor in co-morbidity and health [3]. Complexity involves scenarios where multiple factors from two or more systems (e.g. body-disease, individual-family, patient-provider or provider-organization) influence each other in a dynamic way, producing outcomes that are bound by context [4].

It is the combination of complexity and co-morbidity that presents tremendous challenges for healthcare: increasing amounts of information must be gathered and interpreted in order to understand and manage individual patients’ complex situations, at the same time that patient care must be delivered more rapidly in today’s time-constrained healthcare settings. For this reason, many are seeking more efficient and thorough means of assessing patients’ complex risk profiles. One means of doing so may be through electronic health technologies (eHealth) such as interactive computer-assisted health-risk assessments (HRAs), which aim to improve provider-patient information-sharing and communication in meaningful ways.

HRA tools may be particularly useful in situations involving psychosocial risks, which are both socially sensitive and complex. Several systems operate in their causation, exacerbation and management. Thus, these issues are difficult to broach in time-pressed encounters where patients and providers may not have an established repoire. The utility of HRAs for such encounters is supported by systematic reviews demonstrating higher rates of disclosure of sexual, alcohol, drug, and violent behaviors, as well as HIV status, on computer based surveys than in personal interviews [5-8]. Computers offer a non-judgmental and private mode of self-report with ample time for reflection. In doing so, it offers the space for sense-making about complex conditions in complex environments, which can improve clinical and population health decisions. This study seeks to understand the key factors that influence use and adoption of an interactive HRA, and to explore the potential implications of its use in the prevention and treatment of those with complex conditions.

Building work by Rhodes et al. in the USA [9,10], the research team modeled an interactive HRA for psychosocial health risks. This computer survey focuses on psychosocial issues (e.g., alcohol, tobacco and street drug use, sexual health, conflict in relationships, depression) along with cardiovascular risks to reduce the sensitivity. The tool also assessed income level, food and housing security, language proficiency and social support, in order to provide contextual information essential to forming a realistic healthcare plan. The multi-risk HRA was designed to be completed by patients prior to a medical consultation with a clinician. Once complete, the interactive tool produces printed, individualized risk reports for the clinicians and summary sheets for the patients, both of which include tailored recommendations and contacts of specialist or community-based services.

The interactive HRA was recently examined by the authors in a multi-disciplinary family medicine setting [11], and by Rhodes et al. in an emergency department [12]. These studies demonstrated that, as an eHealth tool, the interactive HRA improved several key aspects of provider-patient communication, namely patient disclosure and provider detection of partner violence and compromised mental health. This is a positive and salient change in clinical practice because both of these psychosocial issues remain under-detected in routine medical visits [13-20] despite high prevalence and serious consequences [21,22]. This under-detection has been linked to patient barriers (e.g., embarrassment and lack of knowledge) and provider constraints (e.g., hesitation and lack of time to inquire) [13-20], both of which may be overcome through use of the interactive HRA.

Although the findings of the aforementioned studies have been positive [11,12] the interactive HRA differs from existing clinical decision-support systems (CDSS) in a number of ways, and the tool would therefore benefit from further research. First, many existing CDSS offer only one-way support to providers through the provision of patient information [23], while the interactive HRA attempted to emphasize humanistic communication in provider-patient dyads by providing feedback to both providers and patients. This is salient due to complexity of psychosocial issues whereby ‘ideal’ approach to resolution is scarce and a patient-centered response is needed. Second, although major medical associations view psychosocial health as an essential component of preventative health [24,25], a recent systematic review reveals that CDSS for preventative care continue to focus on medical conditions (e.g. reminders for cancer screening or vaccination), specific diseases or therapies (e.g., hypertension, diabetes or asthma), physician order entry systems, or preventative care [26]. The interactive HRA, by contrast, is uniquely focused on psychosocial health risks. Third, many existing CDSS fail to capture advanced contextual details of patients’ lives that are important for the assessment of complex and co-morbid conditions, particularly those that are psychosocial in nature (e.g., violence, substance abuse and mental health) [26]. Finally, studies of HRAs have given insufficient attention to the complex healthcare environments into which they are implemented, and the impact the use of HRAs may have on the ways in which providers perform their day-to-day work.

To address some of the aforementioned gaps, the research team initiated an investigation using Concept Mapping method to gather perspectives from a multidisciplinary group of healthcare providers on factors they felt would be essential to the implementation of an effective computer-assisted HRA screening tool for psychosocial risks within their respective clinical practices. The research team drew on the sociotechnical theory to augment the findings [27-29]. This theory views an organization as a system being comprised of social (groups and teams of people) and technical subsystems based on two main tenets. First, the interaction of people and technology in employment settings is viewed as generating not only ‘designed’ linear relationships (i.e., cause and effect) but also ‘un-designed’, non-linear, complex and unexpected relationships. To this end, teams and groups with some primary responsibility but without silo-thinking - *responsible autonomy* – facilitate co-evolution. Second, people and technology in employment settings are regarded as interdependent and, thus, “tinkering” of only one of the two subsystems tends to increase un-designed relationships even dysfunctional ways. It follows that integration and coordination of social and technical subsystems - *joint optimization -* is needed to achieve the desired improvement of the overall system. Theoretically informed advanced understanding of the complex relationships between healthcare providers, administrators, patients and technology is anticipated to facilitate development, implementation and adoption of interactive HRA to manage complex psychosocial health issues.

## Methods

### Study design

Concept Mapping (CM) is a semi-structured qualitative research method. It is participatory in nature and includes three phases: Brainstorming, Sorting and Rating, and Interpretation [30,31]. This method is useful in generating not only the emergent concepts but also for engaging the participants in appraising the relevance of concepts for future planning or direction [32]. The CM method has been used extensively in planning and evaluation [31], and more recently in health studies including healthcare modeling [33,34].

The aim of the Brainstorming phase is to collect a wide range of participant-generated ideas regarding the phenomenon in question, in this case, factors required for the effective implementation of an interactive HRA emphasizing psychosocial health. Brainstorming may be carried out in-person, in individual or group sessions, or via electronic survey. The open approach is useful in capturing a wide range of perspectives from the community of interest. The Brainstorming phase is considered complete when saturation of participant-generated ideas is reached, that is, when no new ideas are being generated. The research team then consolidates the generated statements by eliminating duplicates, very similar statements, and those that appear to be irrelevant.

The refined list of statements is then brought forward to the second phase of Concept Mapping, Sorting and Rating. First, for the sorting activity, participants are asked to sort the brainstormed ideas into groupings that “make sense” for them, and to provide a name for each group. Then, for the rating activity, participants are asked to rate each statement on a Likert-type scale for one or more variables of interest, in this case, relative importance and feasibility. The systematic techniques of sorting and rating are well established in research and add rigor to data collection [35,36]. Like the Brainstorming phase, Sorting and Rating may be carried out either in-person or electronically.

**Figure 1:**
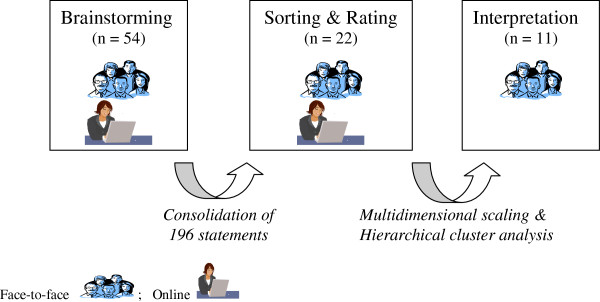
Overview of concept mapping Process.

The research team then performs an analysis of the sorted and rated data using quantitative techniques of multidimensional scaling [37] and hierarchical cluster analyses [38]. This is conducted with the use of a specific statistical package, Concept Systems Incorporated. The statistical analysis generates visual maps which are easy for participants to understand and to evaluate during the Interpretation phase. An overview of the CM phases for this study are presented in Figure 1. While Brainstorming, Sorting and Rating phases may be conducted either face-to-face or via asynchronous on-line sessions, Interpretation requires face-to-face group discussions in order to stimulate response and build consensus.

The research team selected the CM approach for the present study due to the unique mix of qualitative and quantitative techniques employed within a single design. Such a mixed method enables the research project to overcome the methodological weaknesses inherent in individual quantitative designs (e.g., lack of rich descriptions) and qualitative deigns (e.g., lack of analytical rigor or objectivity). Further, the mixed method approach met our transformative paradigmatic stance with the aim of giving voice to the users of emerging technologies.

### Setting and participants

The research team collaborated with an academic hospital serving urban, low income or marginalized populations. Psychosocial health concerns are more prevalent among these underserved populations, so the HRA was especially relevant for healthcare providers working in this setting. A *purposive* sample of 54 healthcare providers was recruited to represent a wide range of disciplines (e.g., nurses, physicians, social workers and managers) from both primary and acute healthcare settings, with the intent of capturing a variety of viewpoints on the “real life” implementation of an interactive HRA tool. The study protocol was approved by the research ethics board of the collaborating hospital (REB # 08–099) and the University of Toronto (REB# 42373).

### Data collection and analysis

The research team provided information about the study to the potential participants by email communication, facilitated by a group of Clinical Managers at the collaborating healthcare institutions. The willing participants expressed their interest directly to the research team. To allow flexibility in participation, the option of face-to-face or online asynchronous data collection was offered for the first two phases. This was critical to engage healthcare providers working nightshifts in the emergency department. Each participant provided an informed consent and completed a demographic survey; an honorarium of $25 was offered for each activity. In the following section, data collection and analytical procedures are described for each phase.

#### Brainstorming

The brainstorming activity was completed by 54 participants; 21 of these completed the activity online; another 33 participated in 3 face-to-face group interviews. Participants were first presented with and asked to review a hypothetical scenario about a multi-risk computer-assisted HRA tool. This was supplemented with a summary of available evidence on the tool’s effectiveness. Participant queries were answered by members of the research team either in face-to-face group sessions or via online communication. Then, participants were asked to respond to an open-ended central question: “*What factors should be in place within your clinical setting to support implementation of an effective computer-assisted screening tool for psychosocial risks?”*

During three separate focus group sessions, brainstormed responses to this question were recorded by a facilitator on a flip chart. Participants who instead took part in the online activity were able to review previously generated responses and to add new responses. In total, 196 individual responses were generated by the 54 participants through the brainstorming activity. These were consolidated by the research team to remove identical or closely similar statements, as well as those that were not relevant to the posed question. This process narrowed the list of factors deemed important for the implementation of computer-assisted screening to 85 statements.

#### Sorting and rating

Twenty-two participants took part in the sorting and rating activities, six in face-to-face interviews and another 15 online. For the sorting activity, each participant was asked to independently review the synthesized statements and to sort them into groups of factors having similar meanings, from his or her own perspective. Participants were then asked to label each group. Participants were instructed to assign each statement to only one group, to refrain from sorting the statements into fewer than three groups, and to refrain from creating groups containing only one statement. For the rating activity, participants rated each individual statement on a scale of 1 (not at all) to 5 (extremely) for two dimensions: its *“importance”* in supporting HRA screening; its *“action feasibility”* within a six-month implementation period. Participant queries were answered by members of the research team through face-to-face or online communication.

#### Visual maps and interpretation

Once the statements were grouped and rated by all 22 participants, the research team analyzed the resulting data using multidimensional scaling. Simple Point Maps were generated wherein each statement was represented as a point on the map. Points which were closer to each other indicated that many people sorted them into similar groups. The stress value for our data set was low (0.26) demonstrating overall good fit [39]. Using hierarchical cluster analysis, the data in the point maps was then used to create Cluster Maps which grouped the points reflecting similar concepts. To identify a cluster configuration where separation or merger of clusters adequately represented the data, the research team began with a 15 cluster solution and increased or decreased the number of clusters by one successively. After reviewing different configurations, a seven-cluster solution was determined to provide the optimal balance between providing sufficient detail and meaningful interpretation. To compare the ratings between importance and feasibility across the clusters, a Rating Bridge map was also created. Examples of these visual maps are discussed below.

All of these maps were shared with the participants who had taken part in the Sorting and Rating activity and joined for the Interpretation sessions. These consisted of two face-to-face focus groups with eleven participants in total. During the groups participants evaluated the cluster content, assessed or modified the cluster labels, provided input into the final cluster solution, and compared the ratings across acute and primary care settings.

## Results

### Participants

Overall, 54 healthcare providers participated in the study (Table 1). Most of the participants were less than 45 years of age (66.7%) and identified as women (85.2%). They included medical doctors (24.1%), nurses (40.7%), social workers (18.5%) and managers/administrators or other allied health staff (16.8%). Participants almost equally represented emergency (48.1%) and family medicine or primary care (51.9%) settings. They worked over 35 h per week (median and mode of 37.5 h). Almost all participants (90%) had clinical experience while management experience was reported by one-third (37%).

**Table 1:** Participant socio-demographics

**Variable**	**Percentage or mean (SD) N = 54**
Age, %	
Less than 35	38.9
From 35 to 45	27.8
From 45 to 55	24.1
More than 55	9.3
Gender, women %	85.2
Professional Training, %	
Medical Doctor	24.1
Registered Nurse/Nurse Practitioner	40.7
Social Worker	18.5
Management/Administration/other	16.8
Clinical Setting, %	
Emergency Medicine	48.1
Family Medicine/Primary Care	51.9
Hours worked per week, mean (SD)	34.8 (9.4)
Clinical Experience, %	
Less than 1 year	3.7
From 1 to 5 years	31.5
From 5 to 10 years	14.8
More than 10 years	40.7
Not applicable	9.3
Management Experience, %	
Less than 1 year	13.0
From 1 to 5 years	7.4
From 5 to 10 years	5.6
More than 10 years	11.1
Not applicable	63.0
Exposure to psychosocial risks in networks, %	
Cigarette smoking	72.2
Alcohol abuse	68.5
Street drug use	38.9
Family violence	35.2
Housing instability	25.9

A summary of key findings and salient aspects within each cluster are presented below.

### Cluster maps and content

**Figure 2:**
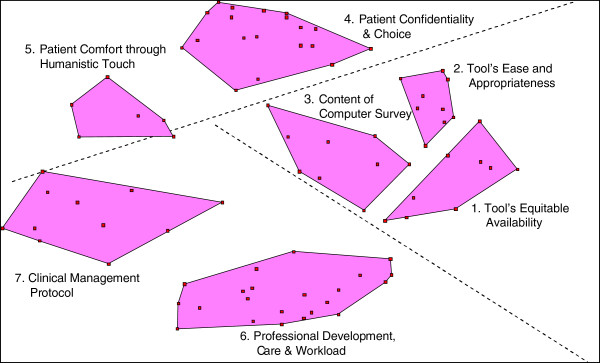
Cluster map.

Seven clusters of statements were ultimately drawn from the 85 responses generated by participants to the question, “*What factors should be in place within your clinical setting to support implementation of an effective computer-assisted screening tool for psychosocial risks?”,* (Figure 2). The cluster names are based on emergent thematic properties of the statements contained within the cluster. Sample statements, also referred to as items, are presented in Table 2. From the ST system theoretical perspective, the seven-cluster map can be seen to have three overarching regions into which the clusters may be grouped (discussed below).

**Table 2:** Clusters: statements and ratings

**Clusters samplestatements**	**Rating mean (SD)†**	
	**Importance**	**Feasibility**
**Technological aspects of HRA forimplementation**		
**Tool’s equitable availability** (9 items)	4.2 (.55)	3.9 (.78)
*… available to both men and women*		
*… available to persons in opposite-sex and same-sex relationships*		
*… location accessible to all, including people in wheelchair*		
**Tool’s ease and appropriateness** (9 items) *	4.5 (.47)	4.1 (.78)
*… questions should be worded in a culturally sensitive manner*		
*… easy to use for patients with no previous use of computers*		
… *have grade 4–6 reading level*		
**Content of computer survey** (9 items)	4.0 (.43)	4.1 (.74)
… *ask about physical and psychological abuse and threats*		
*… ask about mental health issues*		
*… ask about risks for which interventions/resources are available*		
**Social (Patient) aspects of HRA for implementation**		
**Patient confidentiality and choice** (19 items)	4.5 (.32)	4.3 (.62)
*… explicitly inform patient about the purpose of the screening tool*		
*… inform patients about clinician's legal duty to report*		
*… provided patient with a private, safe, and quiet area to complete*		
*… allow patients to skip items they do not want to answer*		
**Patient comfort through humanistic touch** (6 items) *	4.1 (.54)	3.7 (.79)
*… should motivate patients to seek help*		
*… should encourage patients to discuss their risks with providers*		
*… offer assistance and referral in a safe and confidential way*		
**Social (Provider) aspects of HRA implementation**		
**Professional development, care & workload** (22 items) **	4.2 (.37)	3.4 (.92)
*… department specific guidelines on screening inclusion*		
*… prepare clinicians how to respond empathetically*		
*… clearly define the role of all staff*		
*… social workers need to be available 24 h, 7 days*		
**Clinical management protocol** (11 items)	4.0 (.48)	3.8 (.77)
*… clinician's direct immediate contact with patient after disclosure*		
*… make resources (*e.g. *posters, brochures, support staff) available*		
*In family practice, a follow-up visit should be also scheduled*		

†Importance or Feasibility 1–5 Scale: 1 = not at all to 5 = extremely.

Paired *t*-test: * P < 0.05; ** P < 0.001.

The cluster on *Tool’s Equitable Availability* (9 items) included statements pertaining to the technical and developmental aspects of the HRA tool that would enable participation of diverse patients (e.g., physically challenged, differing sexual orientation). The cluster on *Tool’s Ease and Appropriateness* (9 items) contained statements pertaining to the comprehension and clarity of the HRA survey (e.g., non-oppressive language, short and simple, and large font). The cluster on *Content of Computer Survey* (9 items) included items on the types of risks that should be assessed by an interactive HRA tool (e.g., violence and mental health). These three clusters occupied the lower-right region of the map and, are referred to as the Technological aspects of the HRA

The cluster on *Patient Confidentiality and Choice* (19 items) was a large cluster and included items pertaining to informing the patient about the purpose of the assessment, the use and protection of information, the ability of the patient to discontinue the HRA at any time, and the provider’s reporting responsibilities. The cluster on *Patient Comfort through Humanistic Touch* (6 items) contained items pertaining to strategies to promote patient help-seeking for psychosocial issues (e.g., encourage discussions on risks) and prompt responses from providers (e.g., assess immediate concerns and threats). These two clusters occupied the upper region of the map and shared a focus on patients’ interactions with the HRA. This region was referred to as Patient-Social aspects of the HRA.

The largest cluster was *Professional Development, Workload and Care* (22 items), which contained sub-themes on provider training (e.g., empathy and cultural sensitivity where abuse is perceived as a concern), department specific protocols and staff allocation (e.g., decision-tree for clinical management, easy referral to specialists, and 24/7 availability of social workers), and institutional support (e.g., sustainability of the tool’s implementation). A closely located cluster was *Clinical Management Protocol* (11 items), which focused on changes to clinical practice required for appropriate management of disclosure of psychosocial risks (e.g., risk prioritization, resources to make available, follow-up visits, and contacts with referral agencies). These two clusters were in the lower-left region of the map. Through the ST lens, this region revealed an overarching focus on the providers’ interaction with the tool and was referred to as Provider-Social aspects of the HRA.

### Cluster ratings for “importance” and “feasibility”

On a 5-point likert scale, the mean score for the importance of each cluster ranged from 4.0 to 4.5 for the seven clusters. Thus, each cluster was regarded by participants as ‘very important’ for successful implementation of the psychosocial computer-assisted HRA program. The rating pattern was highly similar across the primary care and acute care settings (r = 0.95). Participants’ ratings of the feasibility to act on these clusters within a 6 months implementation period were relatively lower, ranging from 3.4 to 4.3. The rating pattern was moderately similar across the primary care and acute care settings (r = 0.75).

**Figure 3:**
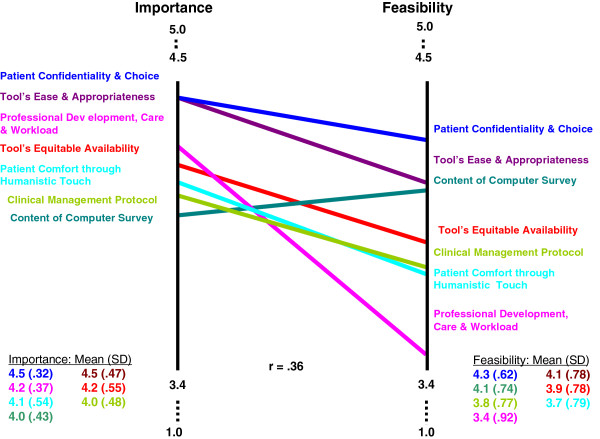
Rating pattern of importance and feasibility scores of clusters.

We employed a paired *t*-test to examine the difference between ‘overall’ scores of clusters for importance and feasibility. The scores were statistically different for clusters of *Professional Development, Workload and Care* (p < 0.001); *Tool’s Ease and Appropriateness* (p < 0.05); and *Patient Comfort through Humanistic Touch* (p < 0.05). Notably, these three clusters represent each of the three regions identified through the ST theoretical lens. Using the visual map for the rating pattern (Figure 3), we asked participants during the interpretation sessions to elaborate on the perceived challenges to implementation of the HRA (discussed below).

### Discussion on feasibility

In terms of *Professional Development, Workload and Care,* participants discussed the need for strong multidisciplinary teams of staff in order to manage complex psychosocial issues in a timely manner. This was perceived as a feasibility challenge particularly in the emergency department setting. Participants discussed financial constraints in having a social worker available 24 h a day in emergency rooms. At the same time, participants felt that a team approach would be essential for overcoming possible delays in provider response to sensitive psychosocial disclosures (e.g. partner violence) in the emergency department. Participants also felt that primary care providers are able to cover a breadth of issues by offering follow-ups, while providers in emergency settings are able to provide only brief interventions. They highlighted the need to have setting specific protocols for clinical management:

R25: Things like immediate direct contact with their clinician after disclosure could be [a] difficult thing to do in [an] emergency room. The worry would be what happens if she disclosed 4 h ago and then couldn’t wait and left, you know, you can’t find them.

The feasibility to act on items in the cluster *Patient Comfort through Humanistic Touch* was perceived as lower for an emergency department than a primary care setting due to the unpredictable nature of patient volume. Further, provision of space and privacy were also perceived as important aspects of provider response to disclosure of psychosocial concerns within emergency department settings.

R6: Well, we have control over the people walking through our door [in family medicine]. In emergency you could have five people or a hundred, so you have no control over the volume coming through.

For the cluster *Tool’s Ease and Appropriateness,* participants felt that the effective implementation of the tool in inner-city clinical settings must attend to the diversity of the patient population. To this end, providers also suggested a step-wise approach to gradually include multiple languages to reach diverse patient groups.

## Discussion

The study findings identify a number of intertwined, multi-level contextual factors that, from the perspective of healthcare providers, must be addressed in order to implement effectively an interactive, computer-assisted HRA with a focus on psychosocial health. Participants viewed the technological and social aspects of introducing a new tool as two sides of a coin, and emphasized each equivocally, although some differences emerged in terms of feasibility. The findings and their implications, both practical and theoretical, are discussed in light of current literature.

The Technological region of the Concept Map included three clusters that focused on the content of the HRA survey and on the ease, appropriateness of and equitable access to the HRA tool. Some of these factors have been previously identified in the existing literature on CDSS, such as the user-friendly features and content of the tool [40-43]. However, providers’ interest in the use of technology to promote patient comfort in general, and equitable access across diverse groups in particular, is unique. This focused attention on patient-related factors may highlight the growing recognition among healthcare providers that, in today’s Internet-based society, patients may be better “informed”, yet will also represent varying degrees of eHealth literacy. In North America, 80% of the general population currently accesses health information on the Internet for themselves, family or friends [44,45]. Moreover, the number of patients bringing Internet-based health information to physicians is on the rise [46,47]. The 2011 results of the Harris Interactive polls report that 57% of the people who go online to search health information discuss it with their doctors [48]. At the same time, a “digital divide” exists in accessing Internet-based information across socioeconomic, age and ethnic groups [49,50]. The participants in our study seemed to view the interactive HRA as having a positive role in reducing this digital divide by offering tailored health assessment and information to their underserved urban patients at the point of care. This suggests that further development of an interactive HRA should offer and evaluate multiple modules (e.g., based on language and literacy, computer literacy, demographic responses, etc.) to respond to patient diversity in eHealth literacy.

The Patient-Social region of the Concept Map centered on patient choice and confidentiality, and on humanistic management of sensitive risk disclosures. This reveals providers’ considerable attention to patient expectations for confidentiality of personal health information on psychosocial risks, and the particular sensitivity to this that may be required when utilizing an interactive HRA. On one hand, this may be a consequence of participants’ anxiety about the medico-legal implications of how sensitive information is handled [13,51]. On the other, it is consistent with providers’ documented lack of confidence in the safe use of eHealth tools. For instance, a recent systematic review of studies on electronic health records identified privacy as an important adoption factor for providers, patients and administrators [43]. Our study findings highlight the ongoing need to engage practitioners in the design and implementation phases of the HRA to enhance providers’ buy-in as well as the responsiveness of the tool for complex health issues.

The Provider-Social region of the Concept Map contained clusters focused on provider training, workload, care, and clinical protocols. This is consistent with existing studies on CDSS, which report the significance of provider training, technical support, work-flow and role definition for successful implementation of CDSS [40-42,52]. While it is widely recognized that the introduction of new technologies impacts how providers perform their day-to-day work of patient care, the impacts and work patterns are continuously evolving. Therefore, ongoing, participatory research with providers utilizing HRA tools is essential in order for gathering real-time, meaningful understanding of the complex contextual factors involved in eHealth implementation.

Notably, participants in our study considered all of the clusters to be equally important for the implementation of computer-assisted HRA. This suggests a readiness of healthcare professionals to embrace the newer eHealth technologies, which focus on psychosocial health risks, in order to connect patients and clinicians and to benefit both simultaneously. For example, Web 2.0 applications, which add to basic World Wide Web functionality elements of interactive information sharing, user centered designs, ability to change website content, and collaboration and co-empowerment of multiple users [53], could enhance interactivity and communication between patients and their clinical teams.

Participants were less sure of the feasibility of implementing the interactive HRA within “the next 6 months”. The Concept Map cluster on *Professional Development, Care and Workload* was rated as posing the greatest challenge, signifying the role of provider buy-in through institutional strategies. Further, one cluster from each of the three regions (i.e., Technological, Social-Provider, and Social-Patient) was judged by participants as presenting greater challenges for feasibility relative to its importance. This suggests that each region is important but not sufficient in itself to ensure successful HRA implementation. In accordance with the *joint optimization* principle of the ST theory, both social and technical aspects of an interactive HRA must be tailored to each health care context into which it is to be implemented in order to ensure success. For example, participants highlighted the differences between acute and primary care settings. This is also supported by the concept of *responsible autonomy* in the ST theory, wherein small groups within an organization facilitate the co-evolution of technology and organization with some primary responsibilities but without silo-thinking. From a practical standpoint, these findings call for attention to multiple interacting contextual factors with a need to develop setting specific-strategies to meet short-term, intermediate and long-terms goals.

The findings of this study also indicate some theoretical contributions. The interconnections between the clusters of statements and their mutual influence on each other suggests that an approach to understanding HRA requires extending the concept of patient-provider interaction towards systems thinking [54-56]. To this end, the ST system theory presents an empirically supported path through emphasis on joint optimization and responsible autonomy. Thus, development of HRA tools that can respond and adapt to changing technological and social circumstances would be likely to meet the needs of the workers and users better than one that assumes a constant state. The ST lens has been widely applied in organizational context for last few decades and now its time to incorporate it in the use of information communication technologies [57].

### Limitations

This is an exploratory qualitative study and the findings present the perspectives of the participants involved. These participants were selected purposively to represent a range of disciplinary perspectives within healthcare. The study recruited healthcare professionals who were working in primary care or acute care units of a teaching-hospital located in inner-city Toronto. The locality of the study, and the unique structure of the Canadian healthcare system, may limit transferability of the findings to other settings, such as smaller cities with lesser population diversity, non-academic settings, and countries with different healthcare structures. The enhanced attention to patient related factors in our study is possibly linked to the underserved patient population typically seen by the participants in this study. There were more female than male participants and clinicians than administrators. This may have limited the inclusion of unique perspectives of male healthcare providers and health administrators. One of the field challenges that the research team observed in this study was the recruitment of healthcare staff from the emergency department. The acute care nature of their work was a barrier to participation in face-to-face group sessions, especially Interpretation sessions for which an online option was unavailable. Some participants commented that the review of 85 statements for the Sorting was time intensive. Further, the Sorting activity through online version was perceived more difficult than the paper-based deck of cards; the computer screens were too small to display all 85 items at the same time. Future concept-mapping studies with busy professionals might need to present a smaller number of items for sorting and rating. Nevertheless, Concept Mapping is a unique approach that combines qualitative collection of data with quantitative statistical analysis and visual maps for participant feedback.

## Conclusion

In conclusion, further development and implementation of the computer-assisted HRA for psychosocial health risks should pay close attention to context-specific social aspects of the technology. To this end, engagement of healthcare workers from clinical settings, such as physicians, nurses, social workers and administrators, is important to assess the ‘weak signals’ that influence rapid system change and provide anticipatory guidance for the HRA and its related service elements [58].

## Competing interests

The authors declare that they have no competing interests.

## Authors’ contributions

FA contributed in the conception and design, acquisition of data, analysis, interpretation and first draft of the manuscript. CN contributed in analysis, interpretation and first draft of the manuscript. PO contributed in the conception and design, analysis and interpretation. All authors read the manuscript for critical revisions and approved the final version.

## Pre-publication history

The pre-publication history for this paper can be accessed here:

http://www.biomedcentral.com/1472-6947/12/149/prepub
